# Shared Ageing Research Models (ShARM): a new facility to support ageing research

**DOI:** 10.1007/s10522-013-9457-0

**Published:** 2013-10-02

**Authors:** Adele L. Duran, Paul Potter, Sara Wells, Tom Kirkwood, Thomas von Zglinicki, Anne McArdle, Cheryl Scudamore, Qing-Jun Meng, Gerald de Haan, Anne Corcoran, Ilaria Bellantuono

**Affiliations:** 1 Department of Human Metabolism, Medical School, Mellanby Centre for Bone Research, University of Sheffield, Beech Hill Road, Sheffield, S10 2RX UK; 2 Medical Research Council Harwell, Harwell Science and Innovation Campus, Oxfordshire, OX11 0RD UK; 3 Newcastle University Ageing Research Laboratories, Institute for Ageing and Health, Campus for Ageing and Vitality, Newcastle upon Tyne, NE4 5PL UK; 4 Faculty of Health and Life Sciences, Institute of Ageing and Chronic Disease, University of Liverpool, 4th Floor, UCD Building, Daulby Street, Liverpool, L69 3GA UK; 5 Faculty of Life Sciences, University of Manchester, AV Hill Building, Oxford Rd, Manchester, M13 9PT UK; 6 European Research Institute for the Biology of Ageing-ERIBA, University Medical Center Groningen, University of Groningen, Antonius Deusinglaan, 1, Building 3226, 9713 AV Groningen, The Netherlands; 7 Babraham Institute, Babraham Research Campus, Cambridge, CB22 3AT UK; 8 MRC-Arthritis Research UK Centre for Integrated Research into Musculoskeletal Ageing (CIMA), Liverpool, Sheffield, Newcastle, UK

**Keywords:** Ageing, Murine models, Tissue bank

## Abstract

In order to manage the rise in life expectancy and the concomitant increased occurrence of age-related diseases, research into ageing has become a strategic priority. Mouse models are commonly utilised as they share high homology with humans and show many similar signs and diseases of ageing. However, the time and cost needed to rear aged cohorts can limit research opportunities. Sharing of resources can provide an ethically and economically superior framework to overcome some of these issues but requires dedicated infrastructure. Shared Ageing Research Models (ShARM) (www.ShARMUK.org) is a new, not-for-profit organisation funded by Wellcome Trust, open to all investigators. It collects, stores and distributes flash frozen tissues from aged murine models through its biorepository and provides a database of live ageing mouse colonies available in the UK and abroad. It also has an online environment (MICEspace) for collation and analysis of data from communal models and discussion boards on subjects such as the welfare of ageing animals and common endpoints for intervention studies. Since launching in July 2012, thanks to the generosity of researchers in UK and Europe, ShARM has collected more than 2,500 tissues and has in excess of 2,000 mice registered in live ageing colonies. By providing the appropriate support, ShARM has been able to bring together the knowledge and experience of investigators in the UK and Europe to maximise research outputs with little additional cost and minimising animal use in order to facilitate progress in ageing research.

## Current challenges faced by researchers using ageing murine models

Social, medical and technological advances, have resulted in the extension of life expectancy and an increase in the number of older individuals (Cracknell 2010). There are many socioeconomic implications of such a change, including the increased occurrence of age-related diseases and the associated rise in health and social care costs (Bloom et al. 2011). In order to find suitable capacity to address these challenges, western countries such as the UK have set ageing as a priority area for their research and development budget (Ageing: Scientific Aspects 2005). The aim is to increase research efforts and target disease prevention, early diagnosis, and discovery of new interventions to ensure people stay healthy for longer.

Central to the successful identification of mechanisms of ageing and age-related diseases is the use of suitable models. Mice are common models of ageing as they share over 95 % homology in gene sequences with humans. Moreover, extensive knowledge has been accumulated on their genetics, which has allowed the generation of very large resources for basic science research and for the generation of disease models. For example, following completion of the mouse genome sequence, an international consortium was developed, the International Knock-out Mouse Consortium (IKMC), to systematically generate mutant embryonic stem cells for every gene in the mouse genome (20,000 plus genes) (Dolgin 2011). In addition, the International Mouse Phenotyping Consortium (IMPC) was formed to coordinate the efforts of carrying out high-throughput phenotyping of each line produced by the IKMC in order to determine the function of every gene in the mouse genome (Brown and Moore 2012). The fact that these mice are being preserved in repositories and made available to the scientific community represents a distinct advantage for using mouse models in ageing research.

However, rearing mice into their old age is expensive. A wild type mouse shows clear signs of ageing and degeneration in most tissues only after 18 months (Ferguson et al. 2003; Sayer et al. 2013). Although costs vary between animal houses, a recent estimate, which considers a commonly accepted mortality rate of 50 % for C57BL/6 mice at 25 months of age (Kunstyr and Leuenberger 1975) set the cost at ~$251/mouse (Conn 2011) and higher for older animals. The high costs, the difficulty in predicting demand on a 2–3 year timescale and the financial implications of unsold animals means only a very small number of animals are commercially available. Consequently, rapid access to aged animals is difficult and ageing research becomes the domain of a small group of well-funded researchers, with the means to support rolling colonies of ageing mice.

Researchers with limited financial means or those newly entering the field of ageing find themselves trapped between the need to maintain the mice for up to 30 months to collect pilot data to apply for funding and the time and high costs that this requires. Most researchers are discouraged and move their research effort elsewhere, limiting the pace of research in the field. In 2010, at the Faculty of Medicine, University of Sheffield a small questionnaire was launched. Out of 26 questionnaires returned, five investigators were already carrying out research into ageing using mice, however an extra eight were not carrying out such research at the time but would have done so if animals were easily available and affordable (Ilaria Bellantuono, personal communication). This suggests there is a potential to increase the number of researchers interested in ageing research if the financial and temporal barriers to acquiring tissues could be overcome. It is with these barriers in mind that ShARM was established to maximise research output.

## The benefits and challenges of establishing a resource for sharing ageing murine models

ShARM is a not-for-profit organisation supporting investigators already undertaking research into ageing and aimed at encouraging others not currently undertaking ageing research to do so, by providing affordable and readily available aged murine tissues through its biorepository and network of live ageing colonies. ShARM also offers a collaborative environment (MICEspace) to create a hub for ageing researchers using murine models and to assist with collaborations.

The major concept behind ShARM was born from the observation that research groups able to maintain ageing colonies are usually interested in a small number of tissues from each mouse and at the time of sacrifice, the surplus tissues are discarded. This is scientifically, ethically and economically ineffective, as the unwanted tissues could potentially be utilised by other investigators. The use of surplus tissues not only benefits investigators in their research efforts but allows simultaneous analysis of multiple organs in a single mouse providing scope for a more comprehensive assessment if the data were to be shared.

The concept is simple but it raises the question, why this has not been done before? A degree of collaborative work had been on-going for years through personal networking and word of mouth. This was very effective for people who had been in the field for a number of years and were well connected but did not work so effectively for new comers to the field. Although the need for such a facility had been discussed for some time in the research community, so were the perceived barriers to success. When ShARM investigators took a closer look at those barriers, it quickly became apparent that there was a need for logistic support and reassurance when sharing information and precious resources. The operative structure of ShARM has been designed to be mindful of these barriers, reducing the burden on investigators.

An important perceived barrier was related to the potential variability of the data obtained from the stored tissues due to the lack of standardization of conditions of housing, diet, pathogens, genetic traits of mice reared in different locations, factors known to affect lifespan (Green 1966). Similarly, investigators were concerned with regard to the quality and the way the tissues were harvested and stored and how these could vary from laboratory to laboratory, introducing variability in the data generated. To address these issues all tissues deposited in the biorepository are collected by the facility coordinator or by selected assistants, trained according to a standard operating procedure (SOP). Procedures were established by the ShARM management board following consultation with pathologists and researchers with expertise in each of the tissues harvested, minimising variability introduced by tissue collection and preservation. Feedback on the quality of the material provided is collected to build trust with the users and ensure a high quality service. As each tissue is barcoded with a unique number, which allows tracking of the animal from which the tissue originated and the animal house, it is possible to identify the source of any problem arising with the quality of the tissues and promptly rectify it. To address the issue of standardization related to the maintenance of ageing mice, large amounts of information including housing, genetic trait, diet, pathogens (see Table 1) are collected with each tissue in a database and passed on to the investigator.

**Table 1:** Information collected with tissues deposited in the biorepository

Contributor info	Mouse info	Cage details	Nutrition	Sacrifice
Anonymous institute ID	Mouse strain DOB Gender Breeder status Source Provision of young, control models Procedure/treatment of animal	Type Size Animals per cage Air changes per hour Frequency of cage change Type of environmental enrichment used	Type of chow Chow composition Chow processing prior to use Feeding regime Watering regime Water type/treatment Water delivery system	Date of sacrifice Time of sacrifice Method used Gross anatomical observations Tissue treatment history

In this way what was once perceived as a barrier has been turned into a tremendous opportunity to collect a vast amount of data which, in time, could be used for meta-analysis to define the impact of those variables on the ageing process. Moreover, it has the potential to allow investigators to produce more robust data by verifying whether the same finding is reproduced using tissues from cohorts of animals reared in different animal houses and all collected according to a standardised procedure. Last but not least, the barcoded database offers the opportunity for further analysis if the investigators are interested to compare data collected on individual tissues (for example “omics” data) obtained from the same animals in the different laboratories. This is dependent on the willingness of investigators to work more collaboratively, requiring a shift in cultural attitudes to sharing and a degree of openness from investigators, who may feel vulnerable to reveal their data, plans or technologies too prematurely. For this reason to ensure investigators feel protected, sensitive data are anonymised and the investigators determine what information is released and when.

A further barrier to the establishment of a facility such as ShARM was the fact that the biorepository would cover only part of the need of researchers as it is no substitute for readily available live animals. However, as rearing animals to old age in large numbers is economically challenging, this requires substantial investment and is not compatible with a self-sustainable facility. For this reason ShARM has established a database of live ageing colonies held by investigators in UK and abroad. This is a system which gives ready access to live mice allowing access to unused fresh tissues which can be collected according to the desired specification. Moreover, it allows greater flexibility in terms of planning for animals to be aged. As research priorities can often change over the time needed to age the mice, ShARM offers researchers the opportunity to communicate with each other and make available any unwanted animals to other researchers, in turn creating greater chances of finding spare aged animals to use at relatively short notice.

## What is ShARM today

ShARM is open to all investigators from academic research institutes and industry. ShARM was initiated by a collaboration between two of the Universities (Sheffield and Newcastle) at the MRC—Arthritis Research UK Centre for Integrated Research into Musculoskeletal Ageing (CIMA) and MRC Harwell. It is supported by Wellcome Trust but has pledged to become self-sustainable within 5 years. The facility is administered in a transparent way through a management board, which includes the investigators holding the Wellcome Trust grant and four users, who serve on the board for 2 years. Any user can apply to become a member of the management board. Balance sheets and minutes of the meetings are available in the members area of the website.

### The biorepository

This is the tissue bank for the acquisition, storage and provision of flash frozen tissues from a range of aged murine models of different strains, ages and sexes. At the time of harvest, unwanted tissues are donated by investigators. Assistance is available for tissue collection or a financial incentive may be available upon request to cover some of the additional costs involved in tissue harvest. Investigators with aged colonies are encouraged to consider depositing samples at the time of harvest. To avoid variability in the collection and ensure quality of the material, all tissues harvested are collected directly by the ShARM coordinator or nominated assistant according to a strict Standard Operating Procedure. At present, the biorepository holds 2,500 tissues from more than 250 mice aged from 1 to 33 months old. Examples of the tissues available for purchase are listed in Table 2. To ensure high tissue quality, the brain and intestine are frozen in liquid nitrogen within 3 mins from the time of sacrifice and all other tissues within 10 mins. Samples are stored at −80 °C in a well-established large scale biorepository which includes forty three freezers with a total capacity of more than 1.5 million samples. The facility is highly secure and monitored 24/7 by an outside control room which is immediately notified if a problem, such as an increase in temperature is detected. At present, it costs £35 for aged tissue and £30 for young controls, excluding shipping costs. These prices are set only to recover costs and may change depending on the costs incurred for the harvest of the tissues.

**Table 2:** Flash frozen tissues available from the ShARM biorepository

Strains	Age range	Tissues available
C57Bl/6J C57Bl/6JBabr C57Bl/6N C57Bl/Icrfa^t^	1–4 and 13–33 months	Bladder Bone (femur, tibia, vertebra) Brain Brown adipose tissue Intestine (duodenum, jejunum, ileum, cecum, colon) Heart Kidney Liver (median, left, right and caudate lobes) Lung Mammary fat pad Muscle Pancreas Prostate Skin Spleen Thymus Thyroid White adipose tissue

Tissue samples are accompanied by a dossier of information regarding the mouse’s pedigree, rearing and method of sacrifice (Table 1). Samples and their data are managed using a highly secure database with standards compliant for the Human Tissue Act regulations for biobanking. The database recognises each individual tissue with a barcode, so samples are easily retrieved without repeated exposure to warmer temperatures and it provides a secure mechanism to connect each sample with the respective data and other tissues derived from the same animal.

On request of the funding body and the commercial suppliers of mice, a material transfer agreement (MTA) is signed every time a tissue is donated or purchased. MTAs are designed to agree the terms of use of the tissues including the division of any intellectual property arising from their use. The MTA establishes that the tissues must be used for in-house research only and cannot be passed on to a third party without the implementation of a subsequent MTA.

### Live ageing colonies

The live ageing colonies comprises an international database of current cohorts of ageing mice from which tissues will be available at the time of harvest. This service provides greater flexibility for acquiring tissues. This may be ideal for investigators who require a specific tissue, not available in the biorepository or require a tissue collected using a specific protocol. Full details of the colonies available can be downloaded from www.ShARMUK.org following registration.

An investigator, interested in using tissues from a colony, will send a request to ShARM’s administrator who will contact the colony owner. Once both parties are agreed, the contact details of both parties will be exchanged and the practicalities of collection can be coordinated. Payment and MTAs are agreed directly between parties. At present the database holds over 2,000 ageing animals.

### MICEspace

MICEspace is an online collaborative environment designed to foster interactions, exchange ideas and knowledge, and provide help and support through forums or private messaging. Opportunities also exist to share best practice especially for carers of the aged mice in order to help improve the welfare and determine humane endpoints.

The facility also offers the opportunity to comprehensively exchange, compare and collate data on a scale unprecedented in the ageing community. Investigators using tissues from communal mice are encouraged to share data which can be analysed to investigate the effect of ageing simultaneously in different tissues.

## Conclusion

ShARM has been designed to provide maximum support to its users and minimise barriers to sharing but its success lies with its users and their spirit of collaboration. Since launching in July 2012, the facility has received surprising enthusiasm with over 50 members registered from six countries and at different stages of their careers. The current economic climate together with increasing demands for sharing by funders and journals may encourage the research community to share, so facilities such as ShARM are timely. Investigators wishing to get involved can visit www.ShARMUK.org and register to become a member. It is a free, quick and easy process which provides access to all the information and means to acquire tissue. Opportunities are also available for members who wish to get involved in the governance of the facility.
